# Heterogeneous Fe_3_ single-cluster catalyst for ammonia synthesis via an associative mechanism

**DOI:** 10.1038/s41467-018-03795-8

**Published:** 2018-04-23

**Authors:** Jin-Cheng Liu, Xue-Lu Ma, Yong Li, Yang-Gang Wang, Hai Xiao, Jun Li

**Affiliations:** Department of Chemistry and Key Laboratory of Organic Optoelectronics & Molecular Engineering of Ministry of Education, Tsinghua University, Beijing, 100084 China

## Abstract

The current industrial ammonia synthesis relies on Haber–Bosch process that is initiated by the dissociative mechanism, in which the adsorbed N_2_ dissociates directly, and thus is limited by Brønsted–Evans–Polanyi (BEP) relation. Here we propose a new strategy that an anchored Fe_3_ cluster on the θ-Al_2_O_3_(010) surface as a heterogeneous catalyst for ammonia synthesis from first-principles theoretical study and microkinetic analysis. We have studied the whole catalytic mechanism for conversion of N_2_ to NH_3_ on Fe_3_/θ-Al_2_O_3_(010), and find that an associative mechanism, in which the adsorbed N_2_ is first hydrogenated to NNH, dominates over the dissociative mechanism, which we attribute to the large spin polarization, low oxidation state of iron, and multi-step redox capability of Fe_3_ cluster. The associative mechanism liberates the turnover frequency (TOF) for ammonia production from the limitation due to the BEP relation, and the calculated TOF on Fe_3_/θ-Al_2_O_3_(010) is comparable to Ru B5 site.

## Abstract (web summary)

The current industrial ammonia synthesis relies on the Haber-Bosch process that is limited by the Brønsted–Evans–Polanyi relation. Here, the authors propose a new strategy that an anchored Fe_3_ on θ-Al_2_O_3_(010) surface serves as a heterogeneous single cluster catalyst for ammonia synthesis from first-principles calculations and microkinetic analysis.

## Introduction

Ammonia synthesis is one of the most important industrial catalytic reactions, which is based on the Haber–Bosch process (N_2_ + H_2_ → NH_3_) since the World War II, and it plays a key role in the growth of human population^1,2^. Although the process using Fe and Ru metal-based catalysts with promoters has been developed for more than one hundred years, it still requires high pressure (~100 bar) and moderately high temperature (~700 K)^3^, which is dictated by the compromise between thermodynamic equilibrium and kinetics^4^. Ammonia synthesis on Fe and Ru metal surfaces is widely regarded as a classical example of correlating the experimentally observable turnover frequency (TOF) with the predicted atomistic mechanism^5^, where the N_2_ activation is confirmed to be a direct N≡N dissociation process^6–8^. The performance of promoted Fe- and Ru-based catalysts clearly shows a site dependence of the N_2_ dissociation and NH_*x*_ desorption^9^. N_2_ molecules are firstly dissociated on specific active sites, such as the B5 site of Ru(0001) step and the C7 site of Fe(111) or Fe(211) surface^5,10,11^, then the adsorbed *N is hydrogenated step by step to produce *NH_3_. This is the well-studied dissociative mechanism.

Previous theoretical studies discussed the possibility of ammonia synthesis at low temperature and low pressure^12,13^, but the TOF was limited, due to the Brønsted–Evans–Polanyi (BEP) relation^14,15^. The BEP relation regulates that the dissociation barrier of N_2_ and the desorption energies of NH_*x*_ scale linearly with the adsorption energy of N atom^12,14,16^. Stronger adsorption of N atom implies lower N_2_ dissociation barrier but higher NH_*x*_ desorption energies, such as on Re, Mo, Fe metal surfaces; while weaker adsorption of N atom indicates higher N_2_ dissociation barrier and lower NH_*x*_ desorption energies, such as on Pd, Co, Ni metal surfaces. Thus a good metal catalyst for ammonia synthesis must have a moderate atomic N adsorption energy, around where the top of volcano plot locates^15,16^.

Several molecular catalysts and naturally occurring nitrogenase enzymes are capable of reducing N_2_ under ambient conditions^17–21^, and the associated mechanisms are likely initiated by the associative adsorption or hydrogenation of N_2_, rather than the dissociative adsorption that is the key to the Haber–Bosch process. Recently, there were some theoretical indications showing that in the electrochemical ammonia synthesis the N_2_ molecule did not dissociate upon adsorption, but was hydrogenated to *NNH instead^22–29^. Even for the thermal catalytic process, Skúlason et al.^25^ showed that the proportion of associative process (i.e., *N_2_ + *H→*NNH + *) is underestimated based on Bayesian statistics. For heterogeneous catalysis, O_2_ and CO can be hydrogenated via an associative mechanism to *OOH and *HCO, respectively, which have been proved to be key intermediates for O_2_ and CO activation^30–36^. When the N_2_ hydrogenation becomes the dominating process, the N–N bond is much weakened, and consequently the dissociation barrier no longer obeys the BEP relation. Thus, designing a catalyst with surface active centers that hydrogenate N_2_ first can be a new strategy to accomplish ammonia synthesis at low temperature and pressure.

The remarkable recent development of surface single-atom catalyst (SAC) and single-cluster catalyst (SCC) demonstrates the possibility to build homogeneous catalytic active centers on heterogeneous solid surfaces^37–41^. Inspired by nitrogenase, in which the FeMoco is responsible for N_2_ activation and ammonia synthesis^42–45^, Holland and co-workers designed a series of multinuclear iron complexes to mimic the nitrogenase, and showed that the formally Fe(I) and Fe(0) complexes can weaken or even break N_2_ triple bond at or below room temperature^18,19,46^. Hosono and co-workers also reported a series of stable electrides as efficient electron donor for loaded Ru metal, which is proved to be more reactive than the bare metal surface^47^.

In this work, we propose an active center of Fe_3_ cluster that is anchored on the θ-Al_2_O_3_(010) surface, and we predict that the direct dissociation of *N_2_ on this center is difficult (dissociative mechanism), but the *N_2_ is easily hydrogenated to form the *NNH species (associative mechanism), which has a much lower N–N bond dissociation barrier than that of *N_2_. We further reveal that the large spin polarization of Fe_3_ is responsible for N_2_ activation, and the low oxidation state iron atom works as an electron reservoir, regulating the charge variation of the whole process. The surface-anchored Fe_3_ SCC renders a robust multi-step redox capability necessary for ammonia synthesis from dinitrogen.

## Results

### N_2_ adsorption on supported Fe_3_ cluster

The FeMoco site of nitrogenase has been considered responsible for biological nitrogen fixation. Its performance in activating N_2_ molecule originates from the highly efficient redox cycle between Fe(II) and Fe(III) of the Mo-Fe-S-C cluster of FeMoco. Based on the model of FeMoco, various Fe containing complexes are designed to mimic the N_2_ activation process on FeMoco^19,20,44,48–51^. A general implication for designing such complex is to keep the Fe center at a reduced state to facilitate electron donation to the N_2_ molecule.

**Fig. 1:**
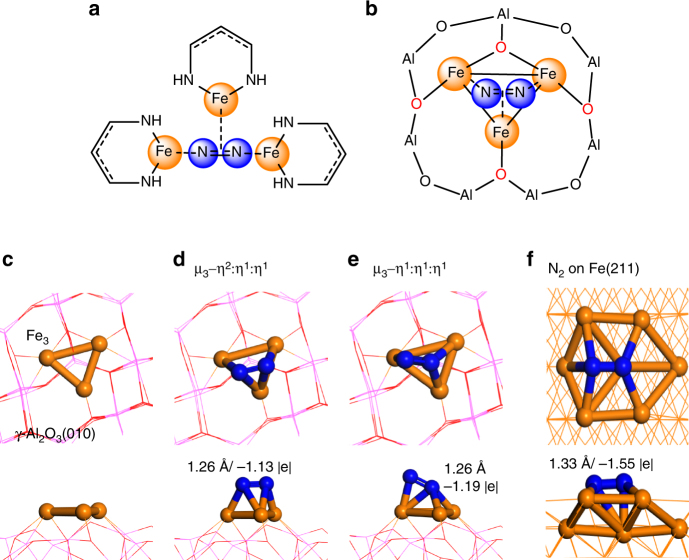
Homogeneous and heterogeneous Fe_3_ cluster with N_2_ adsorption. **a** Schematic representation of N_2_ coordinated with three Fe(I)-ion homogeneous complexes in the side-on/end-on/end-on (μ_3_−η^2^:η^1^η^1^) configuration; **b** schematic representation of N_2_ coordinated with heterogeneous Fe_3_/θ-Al_2_O_3_(010) in the same configuration; **c** optimized Fe_3_ cluster on θ-Al_2_O_3_(010); **d**, **e** optimized configurations of N_2_ adsorption on Fe_3_/θ-Al_2_O_3_(010); **f** N_2_ adsorption configuration on the C7 site of Fe(211) surface

In recent years, embedded Fe clusters with low oxidation state, such as three diketiminate-bound Fe(I) ions in Fig. 1a, are shown to synergistically facilitate N_2_ reduction^18,19,46,48,52^. Inspired by this finding, we conceive that the small Fe clusters supported by θ-Al_2_O_3_(010) surface should be capable of efficient N_2_ activation (Fig. 1b, c), because the inert support has little electronic interaction with the Fe cluster and thus retains Fe in an even more reduced state for metal-metal bonded Fe clusters^3,53^. This type of Fe clusters on alumina surface may be experimentally prepared by soft-landing cluster method or thermal treatment of ligated tri-iron cluster (e.g., Fe_3_(CO)_*n*_)^54,55^. By testing the binding energies for Fe_*n*_ clusters (*n* = 1–5) (Supplementary Fig. 1 and 2), we have shown that the triangular Fe_3_ and the pyramidal Fe_4_ are the most stable clusters on Al_2_O_3_ substrate, with the Fe_3_ cluster kinetically stable against aggregation.

Here we focus on the catalytic performance of Fe_3_ on ammonia synthesis, while we have also proved that the pyramid Fe_4_ cluster, consisting of four triangular planes, exhibits similar catalytic activity to that of Fe_3_ cluster (Supplementary Fig. 3). Fe_3_/θ-Al_2_O_3_(010) with magnetic moment of 10 μB is the most stable, and the state of 8 μB is only 0.08 eV less stable (Supplementary Fig. 4), which is consistent with the *ab initio* molecular dynamics (AIMD) simulations in which the magnetic moment oscillates between 10 and 8 μB (Supplementary Fig. 5g). Such large spin polarization on Fe sites is thought to be one of the key factors to activate N_2_ on FoMoco and Fe complexes^19,43,56^.

Two of the most stable configurations of N_2_ adsorption on the Fe_3_ site are shown in Fig. 1d and e, which are denoted as μ_3_−η^2^ːη^1^ːη^1^ (side-on/end-on/end-on) and μ_3_−η^1^ːη^1^ːη^1^ (end-on/end-on/end-on), respectively. These two configurations are also observed in the AIMD simulations (Supplementary Fig. 5). N_2_ can also adsorb at single or double Fe sites as μ_2_−η^2^ːη^1^, μ_1_−η^1^, and μ_1_−η^2^ configurations^57,58^, but these are less stable here (Supplementary Fig. 6). The Bader charge on N_2_ decreases to −1.13 |e| with its bond length significantly stretched from 1.10 Å in gas phase to 1.26 Å, close to that in N_2_F_2_. In comparison, the N–N bond length on C7 site of Fe(221) surface with high surface energy is 1.33 Å with Bader charge of −1.55 |e| (Fig. 1f).

### Competition between dissociative and associative hydrogenation

Lots of efforts have been made to investigate the mechanism of ammonia synthesis on metal surfaces. It is found that the N_2_ molecule dissociates directly at specific sites of the surface, such as the B5 site of Ru(0001) step surface and the C7 site of Fe(111) or Fe(211) surface^5,10,59^. The barrier of N_2_ direct dissociation over close-packed Ru(0001) surface is as high as 1.9 eV but lowers to 0.4 eV on the step site^6^. Different from on the Fe(C7) site, the binding energy of *NH_*x*_ on Ru surface is lower. According to the BEP relation, the dissociation barrier of N_2_ (Δ*G*^≠^(N_2_)) and desorption energies of *NH_*x*_ (Δ*G*(*NH_*x*_)) scale linearly with the adsorption energy of N atom (Δ*G*(*N)) on the same type of sites^16^, so the dependence of Δ*G*^≠^(N_2_) and Δ*G*(*NH_*x*_) on Δ*G*(*N) can be fitted into lines. It is found that the intercepts of such lines for step sites are lower than those for terrace sites, but the slopes are similar for different kinds of sites^16^. Thus, with the N_2_ dissociative mechanism on metal surfaces (the slopes of Δ*G*^≠^(N_2_) and |Δ*G*(*NH_*x*_)| against |Δ*G*(*N)| are of opposite signs), high temperature and high pressure are intrinsically needed to overcome Δ*G*^≠^(N_2_) and ensure the desorption of NH_*x*_ simultaneously^12,14^.

**Fig. 2:**
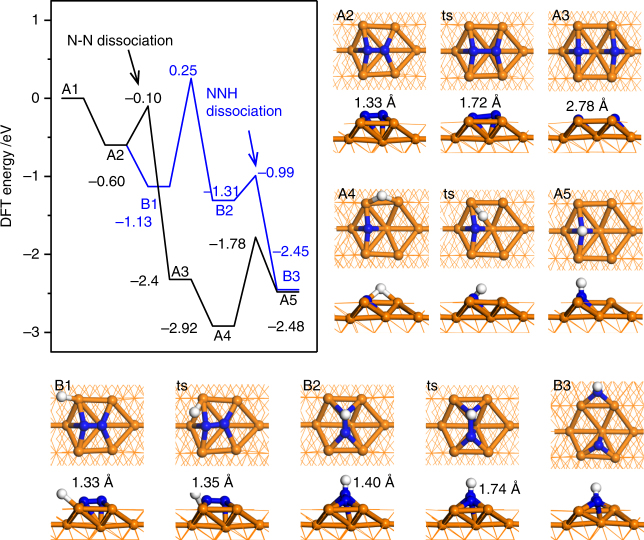
The energy diagram for the first two steps of NH_3_ synthesis on Fe(211) C7 site. The black line is for the dissociative mechanism, where *N_2_ dissociates directly, and the blue line for the associative mechanism, where the N–N bond breaks only after hydrogenation to the *NNH species

When N_2_ adsorbs on Fe(211) surface, the anti-bonding π* orbitals of N_2_ interact strongly with Fe metal surface, and each N is coordinated with four surface Fe, so N_2_ cannot be hydrogenated without reconstruction. As shown in Fig. 2, the *NNH species (B2) has to rotate from the original *N_2_ (B1), with the N–N bond length increased to 1.40 Å. Although the dissociation barrier of *NNH (i.e., from B2 to B3) is only 0.32 eV, the hydrogenation barrier of *N_2_ (from B1 to B2) is as high as 1.38 eV, which is much higher than the *N_2_ direct dissociation barrier. Thus, it is the high hydrogenation barrier initiating the associative mechanism that makes the dissociative mechanism dominate on Fe(211) surface.

**Fig. 3:**
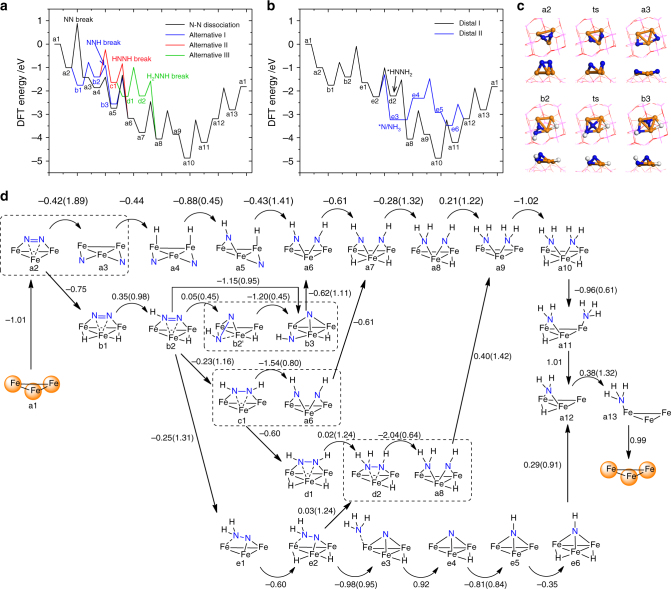
Energy diagrams for ammonia synthesis. **a** The dissociative mechanism, and three pathways of associative mechanism with N–N bond dissociation at *NNH, *HNNH, and *HNNH_2_ intermediates by the alternating hydrogenation route. **b** Two pathways of associative mechanism by the distal hydrogenation route. **c** Initial, transition, and final states of *N_2_ and *NNH dissociation step. **d** Schematic depiction of the six reaction pathways for conversion of N_2_ to NH_3_ catalyzed by Fe_3_/θ-Al_2_O_3_(010). Reaction energies are shown for every step and barriers are enclosed in brackets. The dissociation steps of *N_2_, *NNH, *HNNH, and *HNNH_2_ intermediates are enclosed with dashed lines

Based on the mechanism on metal catalysts, we investigate the reaction pathways of N_2_ activation on single-cluster catalyst Fe_3_/θ-Al_2_O_3_(010), as shown in Fig. 3 and Supplementary Fig. 8. The calculated barrier of *N_2_ direct dissociation is 1.89 eV (Fig. 3c, d), but the associative hydrogenation of *N_2_ to *NNH only has a barrier of 0.98 eV, which can be driven over with relatively low temperature. Note that the H_2_ dissociative adsorption barrier is as low as 0.05 eV (Supplementary Fig. 9), which can be neglected comparably. The *N_2_ dissociation is a structure-sensitive process, which usually needs more than 5 surface metal atoms’ cooperation. The geometry of Fe_3_ cluster is similar to a close-packed Fe(111) surface, where the dissociation of *N_2_ is not favored. However, the electronic structure of supported Fe_3_ cluster is distinct from that of metal surface, which will be discussed in the electronic structures section. On metal surface sites, such as Ru B5 site, the barrier of *N_2_ hydrogenation is about 0.2 eV higher than that of *N_2_ dissociation, which leads to around three orders of magnitude difference between the rate constants^25^. On the contrary, on Fe_3_/θ-Al_2_O_3_(010), the barrier of *N_2_ hydrogenation is 0.91 eV lower than that of *N_2_ dissociation, and the calculated rate constants are 9.8 × 10^5^ s^−1^ and 5.2 × 10^−1^ s^−1^ at 700 K and 100 bar, respectively.

### Reaction mechanisms

As is shown earlier (see also the movie file in the supplementary material), N_2_ is first activated on Fe_3_ active center followed by attacking by dissociated H atom, which differs from the procedure in electrochemical condition where proton attacks the adsorbed N_2_ in solution. With thermochemical condition, after the first N_2_ hydrogenation step, it is more favorable for *NNH to dissociate into *N and *NH rather than further hydrogenation to *HNNH or *NNH_2_. Such associative process is believed to occur in the homogeneous catalysis and enzymatic mechanism^44,50,60,61^. As shown in Fig. 3d, the *NNH can migrate from Fe_3_ to the Fe_3_/θ-Al_2_O_3_(010) interface (i.e., b2 to b2′) with a barrier of 0.45 eV, where the N-end coordinates with two Fe atoms and the NH-end coordinates with one Fe and one substrate Al ion. The dissociation barrier of *NNH in b2′ is only 0.45 eV with an exothermic reaction energy of −1.20 eV (Fig. 3c, d). Afterwards, the upper *N atom moves to the Fe_3_ 3-fold site, while the lower *NH is anchored at the Fe_3_/θ-Al_2_O_3_(010) interface by coordinating with one surface Al ion and two Fe atoms.

The *NNH can also be hydrogenated further via the alternating or the distal pathway^49,51,56^. In the alternating pathway, *NNH is hydrogenated to *HNNH^43^ with a barrier of 1.16 eV (b2 to c1), and the N–N bond length is elongated to 1.42 Å with the N–N stretching frequency of 1012 cm^−1^, suggesting a single bond between N atoms. The formed *HNNH species can either dissociate into two *NH group or be hydrogenated to *H_2_NNH that can again dissociate into *NH_2_ and *NH. The *NH_*x*_ (*x* = 1–2) species can be ultimately hydrogenated to *NH_3_.

In the distal pathway, *NNH is hydrogenated to *NNH_2_ where both H atoms are on the same end of N–N bond. This process requires a barrier of 1.31 eV, slightly higher than that of the alternating pathway. Next, *NNH_2_ can be further hydrogenated to *HNNH_2_ or *N + *NH_3_. The latter step features spontaneous N–N bond dissociation with a barrier of 0.95 eV, while the former step drives the distal pathway back to the alternating one with a barrier of 1.24 eV.

Overall, we find that the most hydrogenation steps experience barriers ranged from 1.0 to 1.3 eV, which are close to those on metal surfaces^5,6^, but the indirect N–N bond breaking via *NNH has only a barrier of 0.45 eV, much lower than the direct *N_2_ dissociation. As a result, the N–N bond breaking step is not the rate-determining step (RDS) of ammonia synthesis on surface-supported Fe_3_ cluster, distinct from on the traditional metal catalyst surfaces.

The N–N bond dissociation via *NNH bypasses the direct dissociation of *N_2_ that is one of the severe limitations for the Haber–Bosch process. On the Sabatier volcano curve^14^, the *N_2_ dissociation is replaced by the *NNH dissociation, which has a lower barrier that can be driven over with moderate thermodynamic conditions. Therefore, the RDS is no longer the dissociation of N_2_ but desorption of NH_*x*_ species. Such an associative mechanism for N_2_ activation is what nitrogenase does in nature^43^, and has been reproduced by metal complexes for homogeneous catalysis, where the N_2_ triple bond is first weakened by single-metal or multiple-metal center, and then hydrogenated by protons and electrons toward NH_3_ formation^42^. The surface-anchored Fe_3_ center with multi-step redox capability, large spin polarization and low oxidation state metal provides a heterogeneous single-cluster catalyst design to mimic the FeMoco in nature.

**Fig. 4:**
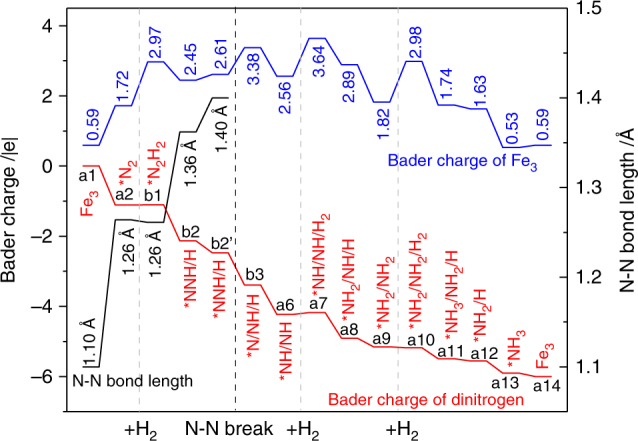
Changes of N–N bond length and charges of Fe_3_ and the adsorbates. Black, blue, and red curves represent N–N bond lengths, Bader charges of Fe_3_ cluster, and Bader charges of dinitrogen at every step of the catalytic cycle with the associative mechanism

We further tracked the Bader charges of Fe_3_ cluster and the N-containing adsorbates along the reaction pathway with associative mechanism, as shown in Fig. 4. The highly reducing Fe_3_ cluster is strongly oxidized during the whole process. When N_2_ and H_2_ are adsorbed on Fe_3_ cluster, the Bader charge of Fe_3_ increases from 0.59 to 2.97 |e|, while N_2_ and H_2_ are reduced to *N_2_^–^ and 2*H^–^ adsorbates. The bond order of N_2_ is reduced to 2.5, with its bond length of 1.26 Å and stretching vibrational frequency of 1384 cm^−1^. Then, one *H^−^ combines with *N_2_^–^ to form a *NNH^–^ species, with one electron released back to Fe_3_ simultaneously. The N–N bond length is then lengthened to 1.36 Å with stretching frequency of 1099 cm^−1^, and the N–N bond order becomes 2.0 with around two electrons occupying its π* orbitals. Bader charges on Fe_3_ and *NNH turn to 2.45 and −1.13 |e|, respectively. When *NNH migrates from the Fe_3_ cluster to the Fe_3_/θ-Al_2_O_3_(010) interface, the N–N bond length is further stretched to 1.40 Å with Bader charge of −1.47 |e|. As shown in Fig. 4, Fe_3_ is a bifunctional multi-step redox active center for donating electrons at the adsorption steps and accepting electrons at the hydrogenation steps. Thus, the surface-anchored Fe_3_ single cluster serves as an electron reservoir that regulates the charge variation of the whole process.

### Electronic structures

**Fig. 5:**
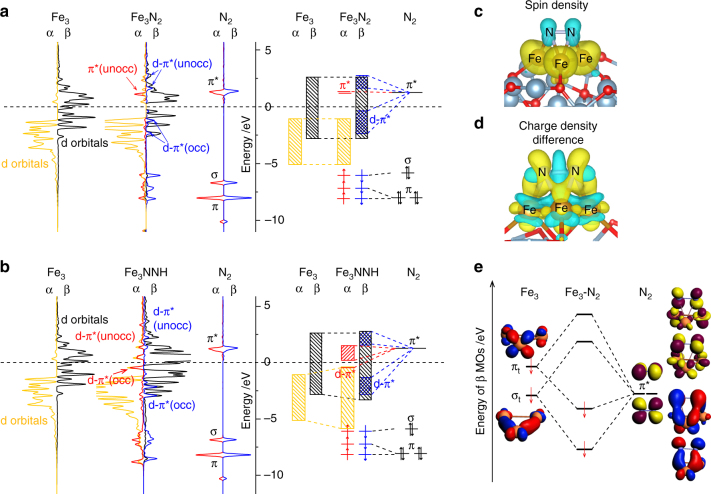
Electronic structure analysis. **a** Projected electronic densities of states (pDOS) and schematic illustrations of 3d orbitals of Fe_3_ cluster on θ-Al_2_O_3_(010), 2p-orbitals of the N_2_ gas molecule, and their interaction within Fe_3_N_2_/θ-Al_2_O_3_(010) of μ_3_−η^2^:η^1^η^1^ configuration. **b** pDOS and schematic illustrations for the Fe_3_NNH/θ-Al_2_O_3_(010) case. **c** Spin density of Fe_3_N_2_/θ-Al_2_O_3_(010). (yellow stands for spin up and cyan for spin down) **d** Charge density differences (*δ*ρ = *ρ*_A+B_−*ρ*_A_−*ρ*_B_) of N_2_ adsorption on Fe_3_/θ-Al_2_O_3_(010) (cyan stands for holes and yellow for electrons). **e** The major interactions and energy levels of the scalar relativistic Kohn–Sham β-spin MOs of isolated Fe_3_N_2_ with correlation to the orbitals from Fe_3_ and N_2_ fragments

To elucidate the bonding nature of the species involved in the associative mechanism, we investigate the densities of states (DOS) of N_2_ adsorption on Fe_3_/θ-Al_2_O_3_(010) (with the most stable configuration μ_3_−η^2^ːη^1^ːη^1^) and C7 site of Fe(211) (Fig. 5a and Supplementary Fig. 10) for comparison. The energy levels of Fe_3_ minority β-spin d orbitals and N_2_ π* orbitals are well matched, leading to partial occupation of the formed d-π* orbitals. While the strong spin polarization provides large exchange stabilization energy for the majority α-spin orbitals, leading to that the energy levels of Fe_3_’s α-spin d orbitals are about 2.5 eV lower than the π* orbitals of N_2_, and thus no obvious interaction of α orbitals is observed. This indicates that only the β π* orbitals of *N_2_ are partially occupied, and thus forcing *N_2_ to be of radical nature (with unpaired electron, Fig. 5c) that is active for hydrogenation. Therefore, the large spin polarization on Fe_3_ cluster is responsible for the activation of N_2_. When *N_2_ is hydrogenated to *NNH, one electron transfers from hydrogen to the α π* orbitals of *N_2_, and now both α and β DOS of Fe_3_’s d orbitals overlap with NNH’s π* orbitals, which further weakens the N–N bond. Therefore, the more interaction and thus larger occupation of the π* orbitals in NNH leads to a lower N–N bond order, and is thus responsible for the lower dissociation barrier than that of *N_2_.

Fragment orbital analysis is further performed to provide more details of such interaction between isolated Fe_3_ and N_2_ (Fig. 5e, Supplementary Fig. 11 and Supplementary Table 1). The main contribution to the interaction is from the tangential σ_t_, π_t_ molecular orbitals of Fe_3_ and N_2_ π* orbitals, which lead to two bonding orbitals and two anti-bonding orbitals. This bonding model shows that the electrons from metal d orbitals partially transfer to the empty π* orbitals of N_2_, which is consistent with the electron transfer shown in the charge density difference (Fig. 5d) and the DOS analysis. Remarkably, in homogeneous catalysis, the N_2_ activation process is also initiated with the electron transfer from the electron-rich metal center to the empty π* orbitals of N_2_, which lowers the bond order in N_2_ and facilitates the hydrogenation process.

### Microkinetic simulations

**Fig. 6:**
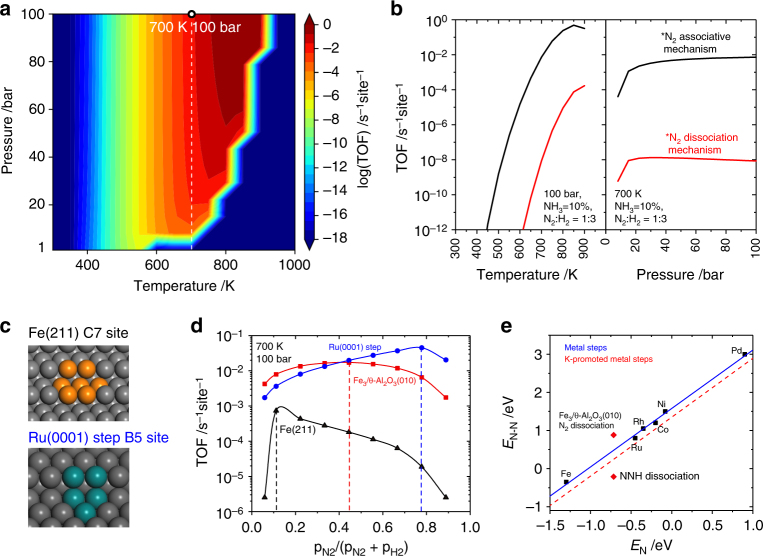
Microkinetic simulations. **a** TOF per site of ammonia synthesis on Fe_3_/θ-Al_2_O_3_(010) mapped with pressure (1–100 bar) and temperature (300–1000 K). H_2_:N_2_ ratio is fixed at 3, and the conversion ratio of NH_3_ is 10%. **b** TOF contributions from the associative mechanism (black curve) and the dissociative mechanism (red curve) at constant pressure of 100 bar and constant temperature of 700 K, respectively. **c** Structures of B5 site on Ru(0001) step surface and C7 site on Fe(211) surface. **d** TOFs per site of ammonia synthesis over the three catalysts as a function of N_2_ partial pressure at 700 K and 100 bar. **e** Calculated transition state energies (*E*_N–N_) for N_2_ dissociation or NNH dissociation as a function of the nitrogen adsorption energy (*E*_N_) over step sites on Fe, Ru, Rh, Co, Ni, and Pd surfaces. The solid blue line represents a least-squared interpolation between the points, the red line depicts the cases with addition of alkali (potassium) promotion. Note that the energy data for the metal surfaces in **e** are taken from ref. [12]

To explore the reactive performance of ammonia synthesis under realistic conditions, we performed comparative kinetic analysis of Fe_3_/Al_2_O_3_, Fe surface, and Ru surface based on the free energy calculations. (Supplementary Fig. 12 and 13) The TOF map (Fig. 6a) is calculated under the pressure range of 1~100 bar and the temperature range of 300–1000 K. The TOF of ammonia production on Fe_3_/Al_2_O_3_ is less than 10^−10^ s^−1^ site^−1^ below 400 K because of too stable adsorption of NH_*x*_ species (Supplementary Fig. 14a). At high temperature and low pressure, the conversion of NH_3_ is lower than 10%, and the decomposition of ammonia occurs. With the increase of temperature, the concentrations of NH_*x*_ decrease and that of bare site increases, since the entropy of free gas molecules becomes dominant. The calculated TOF of ammonia synthesis on Fe_3_/Al_2_O_3_ is 1.4 × 10^−2^ s^−1^ site^−1^ at 100 bar and 700 K, which is comparable to that on the Ru B5 site^12^. As shown in Fig. 6b, the contribution from the associative mechanism is six orders of magnitude larger than from the dissociative mechanism. While on the Ru step surface, the TOF of the associative mechanism is calculated to be three orders of magnitude lower than that of the dissociative mechanism^25^.

Based on the above microkinetic analysis of the ammonia synthesis on Fe_3_/θ-Al_2_O_3_(010), we further compare it with the widely reported cases on Fe C7 site and Ru B5 site (Fig. 6c)^5,6,12,62^. The coverages and free energy diagrams for surface species are shown in Supplementary Fig. 13 and 14. For Fe C7 site, although the transition state energy of *N_2_ dissociation is only −0.10 eV with respect to the gas phase N_2_ and clean surface, the adsorption energy of two *N is as high as −2.4 eV (Fig. 2), which results in dominant coverage of *N on the active center (Supplementary Fig. 14f). Thus, the RDS on Fe C7 site is the desorption of *NH_*x*_ species. For Ru B5 site, the transition state energy of *N_2_ dissociation is 0.4 eV, with two *N adsorption energy of −0.8 eV. At low (<450 K) and high (>450 K) temperature, the Ru B5 site is covered by *NH_2_ and *H, respectively. Thus, Ru B5 site is the closest to the top of volcano curve, which balances the *N_2_ dissociation and *NH_*x*_ desorption processes.

For our single-cluster catalyst Fe_3_/θ-Al_2_O_3_(010), the Fe_3_ active site is covered by *NH_2_ and *NH_3_ at low temperature, and at constant temperature or pressure (Supplementary Fig. 14), the TOF on Fe_3_/θ-Al_2_O_3_(010) is comparable to that on Ru B5 site, and is about two orders of magnitude larger than on Fe C7 site. (Supplementary Fig. 15) By plotting the TOF against the partial pressure of N_2_ as shown in Fig. 6d, the maxima for Fe C7, Fe_3_/θ-Al_2_O_3_(010), and Ru B5 site are 0.06, 0.44, and 0.78, respectively. As the Fe C7 site is mainly covered by *N, lowering the N_2_ partial pressure from 25% to 6% increases the TOF. On the contrary, for Ru B5 site, the main surface species is *H at 700 K and 100 bar, and thus increasing the proportion of N_2_ accelerates the reaction. For Fe_3_/θ-Al_2_O_3_(010), to reach a high TOF, one should balance the partial pressure of N_2_:H_2_, because the associative mechanism requires the co-adsorption of N_2_ and H_2_ to form the *NNH species, which is the key intermediate as discussed above.

As shown in Fig. 6e, for most active sites on metal surfaces, there is a linear relation (i.e., BEP relation) between the adsorption energies of N atom (*E*_N_) and the transition state energies (*E*_N–N_, which is equivalent to the apparent activation barrier) for N_2_ dissociation. On Fe_3_/θ-Al_2_O_3_(010), although the *E*_N–N_ is above the line for metal cases, the transition state energy of NNH dissociation is even much lower than the line for potassium-promoted metal cases. It is thus possible to bypass the BEP relation with the associative mechanism, and the limitation underlying one side of the volcano curve is then removed. As a counterpart, very recently Chen et al. showed^63,64^ very interesting cases that the other scaling relation between *E*_N_ and desorption energies of NH_*x*_ can also be broken by nitrogen transfer from metal to LiH, which separates the N_2_ dissociation site from the hydrogenation and desorption site. With these strategies of breaking the BEP relation, ammonia synthesis with ambient conditions does not seem to be impossible. While this study focus on the Fe_3_/Al_2_O_3_ system, it is conceivable that change of support can influence the charge state of the metal cluster and alteration of the transition metal can also affect the N–NH bond breaking barrier. Especially, the procedure of N_2_ activation and hydrogenation can be affected by moisture or solvents if existing. Further study of the optimal surface metal clusters and support for N_2_-to-NH_3_ conversion under different chemical conditions will be interesting. The nature of the surface SCCs will likely offer high selectivity like single-atom catalysts.

## Discussion

We propose a surface-anchored Fe_3_ single-cluster active center for ammonia synthesis by first-principle calculations and microkinetic analysis. This Fe_3_ cluster can be stably anchored on the θ-Al_2_O_3_(010) surface, and its multi-step redox capacity, large spin polarization and low oxidation state metal enable efficient N_2_ activation, due to the spin-polarized charge transfer from Fe’s 3d orbitals to N_2_ π* orbitals. The partial occupation of N_2_’s β-spin π* orbitals both lowers the N–N bond order and also grants *N_2_ a novel radical nature that leads to an associative mechanism for N_2_ activation, which mimics the initiation process in the nitrogenase as well as artificial metal complexes for homogeneous catalysis involving nitrogen fixation.

We predict the whole catalytic mechanism for ammonia synthesis on Fe_3_/θ-Al_2_O_3_(010) that is distinctly different from on the industrially used Fe and Ru metal surfaces. The dissociative mechanism dominates the ammonia synthesis on metal surfaces, where *N_2_ dissociates directly. Thus, the TOF of ammonia synthesis on metal surfaces obeys the BEP relation that demands the balance between N_2_ dissociation and NH_*x*_ desorption. However, we find that in our associative mechanism at the single-cluster catalyst the first hydrogenation of N_2_ to *NNH is much faster than the dissociative mechanism on Fe_3_/θ-Al_2_O_3_(010), and the following dissociation of *NNH only has a barrier of 0.45 eV. Remarkably, the associative mechanism bypasses the BEP relation and thus the limitation underlying one side of the volcano curve. Such surface-anchored Fe_3_ center represents a class of new catalyst—single-cluster catalyst, which features identical yet isolated active centers on support and thus bridges the gap between heterogeneous and homogeneous catalysis, serving as a heterogeneous catalyst design that enables the associative mechanism for ammonia synthesis from dinitrogen.

The calculated TOF of ammonia synthesis on Fe_3_/θ-Al_2_O_3_(010) is comparable to that on Ru B5 site, which is known as the most active metal catalyst, and is two orders of magnitude faster than Fe C7 site. Thus, the anchored Fe_3_ center is a promising candidate heterogeneous catalyst for highly selective ammonia synthesis via the associative mechanism. In the future work, we will conduct extensive investigation of various surface-anchored metal trimer and polynuclear clusters, in order to find the optimum of such design for ammonia synthesis toward high TOF at low temperature and low pressure. Highly stable single-cluster catalysts with well-accommodating support hold promises for rational design of highly selective and active catalysts for complicated catalytic reactions such as N_2_-to-NH_3_ conversion.

## Methods

### DFT parameters

Energetics calculations for reaction mechanisms were carried out by using spin-polarized density functional theory (DFT) with Perdew-Burke-Ernzerhof (PBE)^65^ generalized gradient approximation as implemented in VASP 5.3.5^66^. The cutoff energy of plane-wave basis set is 400 eV and single gamma-point grid sampling was used for Brillouin zone integration. Atomic positions were optimized until the forces were less than 0.02 eV/Å. Transition states were searched by climbing image nudged elastic-band method (CI-NEB) and further confirmed by vibrational frequency analysis^67^.

### Computational model

A θ-Al_2_O_3_(010)-p(2 × 4) surface slab was used to model the substrate with cell parameters of a = 11.24 Å, b = 11.79 Å, and c = 25.00 Å. The slab consists of seven O−Al layers, where the bottom two O−Al layer were frozen while the remaining layers were allowed to relax. The slab lattice parameters were fixed to the optimized cell parameters of bulk θ-Al_2_O_3_, in order to mimic the support bulk. The trinuclear Fe clusters were anchored by coordinating with surface oxygen as shown in Fig. 1c, and Supplementary Fig. 1. The formation energy of adsorbed iron clusters is defined as *E*_f_ = *E*(Fe_*n*_/Al_2_O_3_)–*E*(Al_2_O_3_)–*nE*(Fe), where *E*(Fe_*n*_/Al_2_O_3_) is the total energy of Al_2_O_3_ surface with Fe_*n*_ adsorbed, *E*(Al_2_O_3_) is the energy of pristine Al_2_O_3_ surface, and *E*(Fe) is the energy per atom of Fe metal. So the formation energy includes both the formation energy of gas phase Fe_*n*_ cluster and its binding energy on surface. Therefore, we can directly evaluate the stability by *E*_f_. The binding energy of Fe_3_ cluster is defined as *E*_bind_ = *E*(Fe_3_/Al_2_O_3_)–*E*(Al_2_O_3_)–*E*(Fe_3_), where *E*(Fe_3_) is the energy of gas phase Fe_3_ cluster. The adsorption energies of molecules are defined as *E*_ads_(X) = *E*(X/Al_2_O_3_)–*E*(Al_2_O_3_)–*E*(X), where X is H_2_, N_2_, and NH_3_. The adsorption energy of N atom is defined as *E*_ads_(N) = 1/2 (*E*(2 N/Al_2_O_3_)–*E*(Al_2_O_3_)–*E*(N_2_)), where *E*(2 N/Al_2_O_3_) is the total energy for N_2_ dissociative adsorption.

### Chemical bonding analysis

Electronic structure analyses were performed using spin-unrestricted DFT with PBE exchange-correlation functional and TZ2P Slater basis sets as implemented in the Amsterdam Density Functional (ADF 2016.101) program^68^. Frozen core approximations were applied to N [1 s^2^] and Fe[1s^2^–2p^6^]. The scalar relativistic (SR) effect was included by the zero-order-regular approximation (ZORA)^69^. Fe_3_ and Fe_3_N_2_ were optimized under D_3h_ and C_s_ point-group symmetries, respectively, at all possible spin configurations. Molecular orbitals (MOs) of Fe_3_N_2_ and their corresponding contributions from Fe_3_ and N_2_ were obtained from fragment MO analyses.

### Microkinetic method

Microkinetic modeling was carried out using the CatMAP software package^70^. The model was constructed by numerically solving the differential equations that describe the coverage of each surface intermediates under the steady-state approximation. The rate constant of each elementary step was calculated by using harmonic transition state theory. The free energies for gas molecules were estimated using the ideal gas approximation considering the vibrational, rotational, and translational contributions to both entropy and enthalpy, while the free energies for surface adsorbates were approximated using the harmonic approximation that treats all degrees of freedom as vibrational modes. The steady-state TOFs were calculated based on the steady-state surface coverages.

### Data avalability

All other data supporting the findings of this study are available in the article and its Supplementary Information files and from the corresponding author on request.

## Electronic supplementary material

Supplementary Information

Descriptions of Additional Supplementary Files

Supplementary Movie 1

Peer Review File
