# Supplementary material for: Heterogeneous Fe3 single-cluster catalyst for ammonia synthesis via an associative mechanism
Source: Nat Commun. 2018 Apr 23;9:1610. doi: 10.1038/s41467-018-03795-8 (PMC5913218; doi:10.1038/s41467-018-03795-8)
Supplement: Supplementary file 1 — Supplementary Information [file 41467_2018_3795_MOESM1_ESM.doc]

**Supplementary Information for**

**Heterogeneous Fe_3_ Single-Cluster Catalyst for Ammonia Synthesis via an Associative Mechanism**

Liu *et al.*


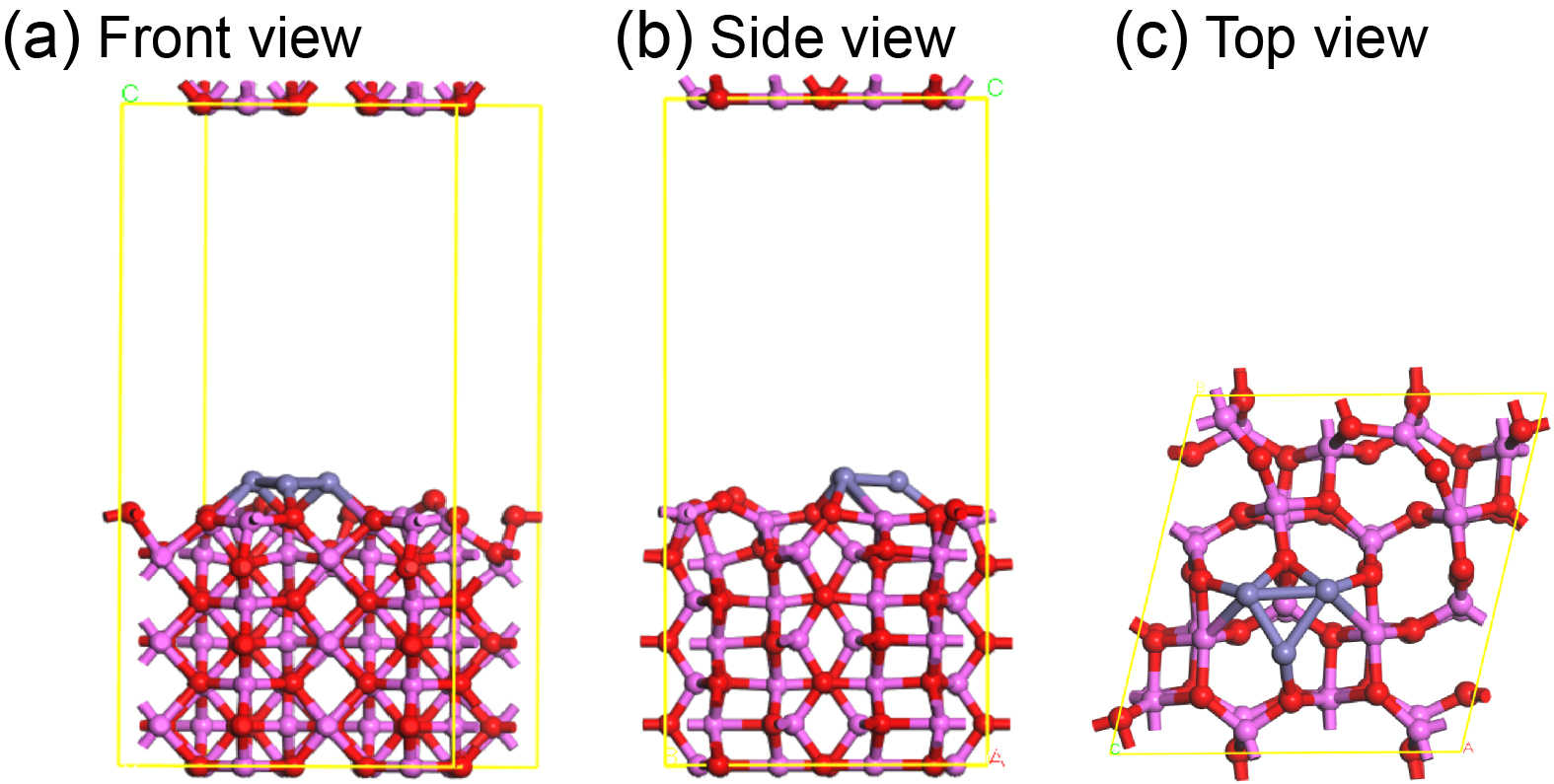


**Supplementary Figure 1**. The optimized most stable structure of Fe_3_/θ-Al_2_O_3_(010). (a) front view, (b) side view, and (c) top view


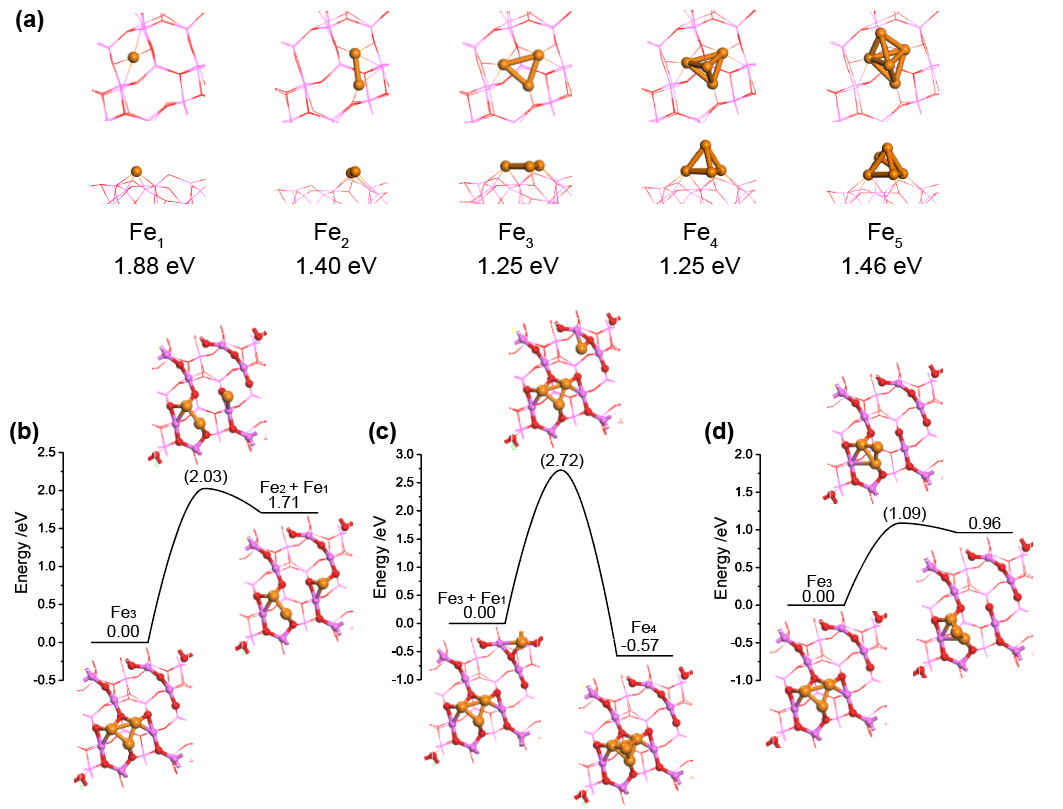


**Supplementary Figure 2.** Stability of Fe_3_ cluster on alumina. (a) Configurations of Fe*_n_* (*n* = 1-5) clusters and the formation energy per iron atom with respect to bulk iron. Energy profiles and corresponding configurations of (b) the dissociation of Fe_3_ to Fe_2_ and Fe_1_, (c) the aggregation of Fe_3_ and Fe_1_ to Fe_4_, and (d) the reconfiguration of Fe_3_ cluster.

We calculated here the barriers and energetics for dissociation of Fe_3_ to Fe_2_ and Fe_1_ (Supplementary Figure 2b), and the aggregation of Fe_3_ and Fe_1_ into Fe_4_ (Supplementary Figure 2c). The calculated barriers for both processes are more than 2 eV. Although the aggregation of Fe_3_ to Fe_4_ is slightly exothermic, the barrier is as high as 2.72 eV, indicating that the Fe_3_ clusters are kinetically very stable on θ-Al_2_O_3_(010) surface. We also tested the reconfiguration of Fe_3_ cluster from the horizontal position to vertical one, and found a barrier of more than 1 eV.


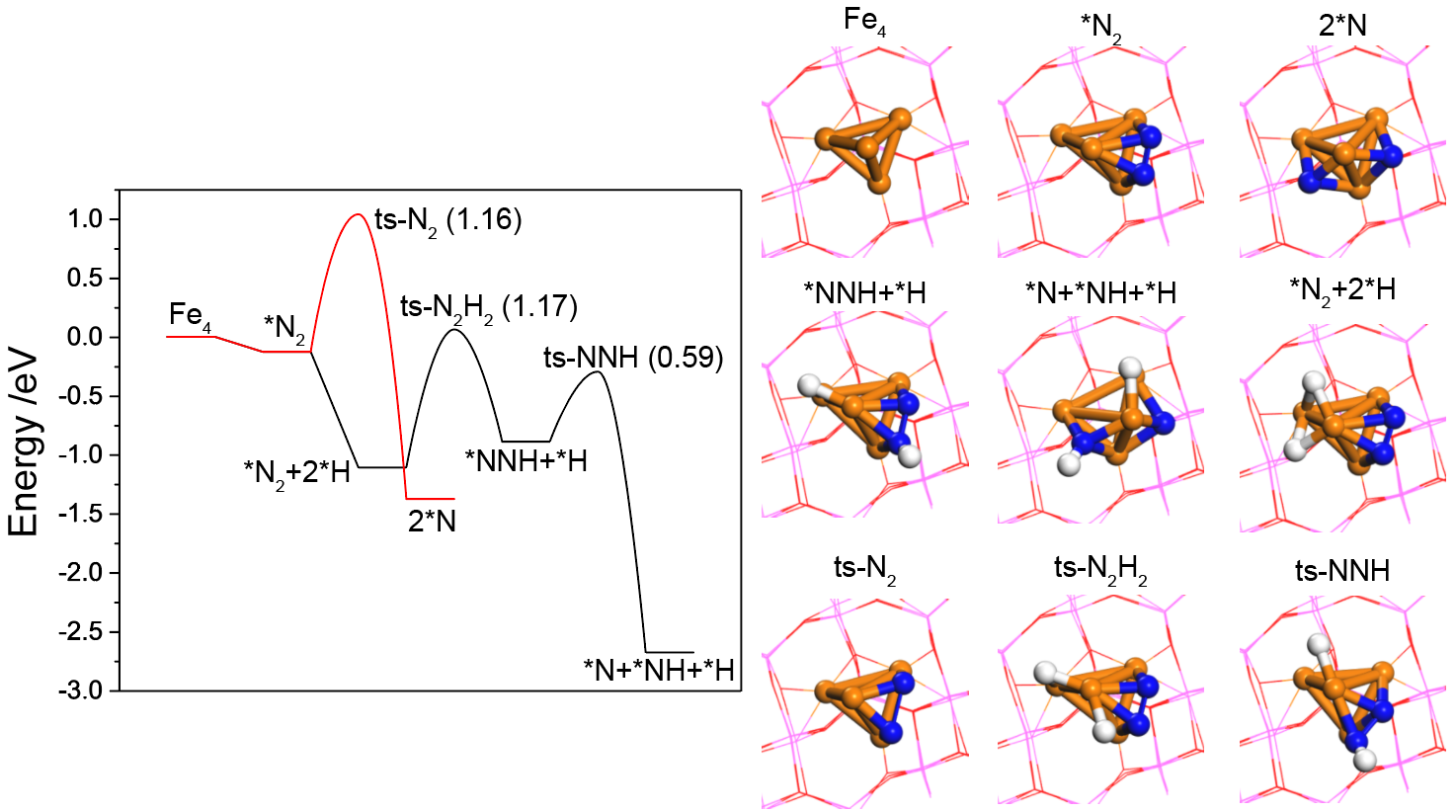


**Supplementary Figure 3.** Energy diagram and structures for the first two steps of ammonia synthesis on the Fe_4_/θ-Al_2_O_3_(010). Red line for the direct dissociation of dinitrogen and black line for the associative pathway with dissociation of NNH.

We have shown that N_2_ could also adsorbs at the three-fold center of Fe_4_ cluster with μ_3_−η^2^:η^1^:η^1^ (side-on/end-on/end-on) configuration. The dissociative barrier of N_2_ is 1.16 eV, which is a little lower than that on Fe_3_ cluster. The barrier of hydrogenation of *N_2_ to *NNH species is 1.17 eV and the following dissociation of *NNH only need to overcome a barrier of 0.59 eV, which is similar as the associative pathway on Fe_3_ cluster.


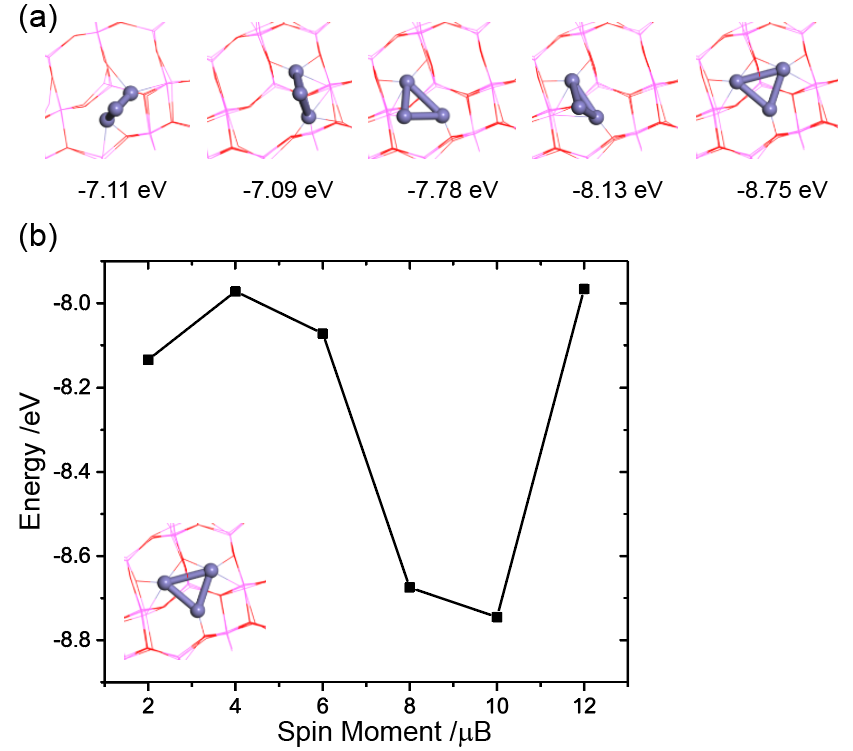


**Supplementary Figure 4**. Structures and binding energies. (a) optimized structures and corresponding binding energies of supported Fe_3_ cluster on θ-Al_2_O_3_(010). (b) Fe_3_ binding energies at various spin moment.


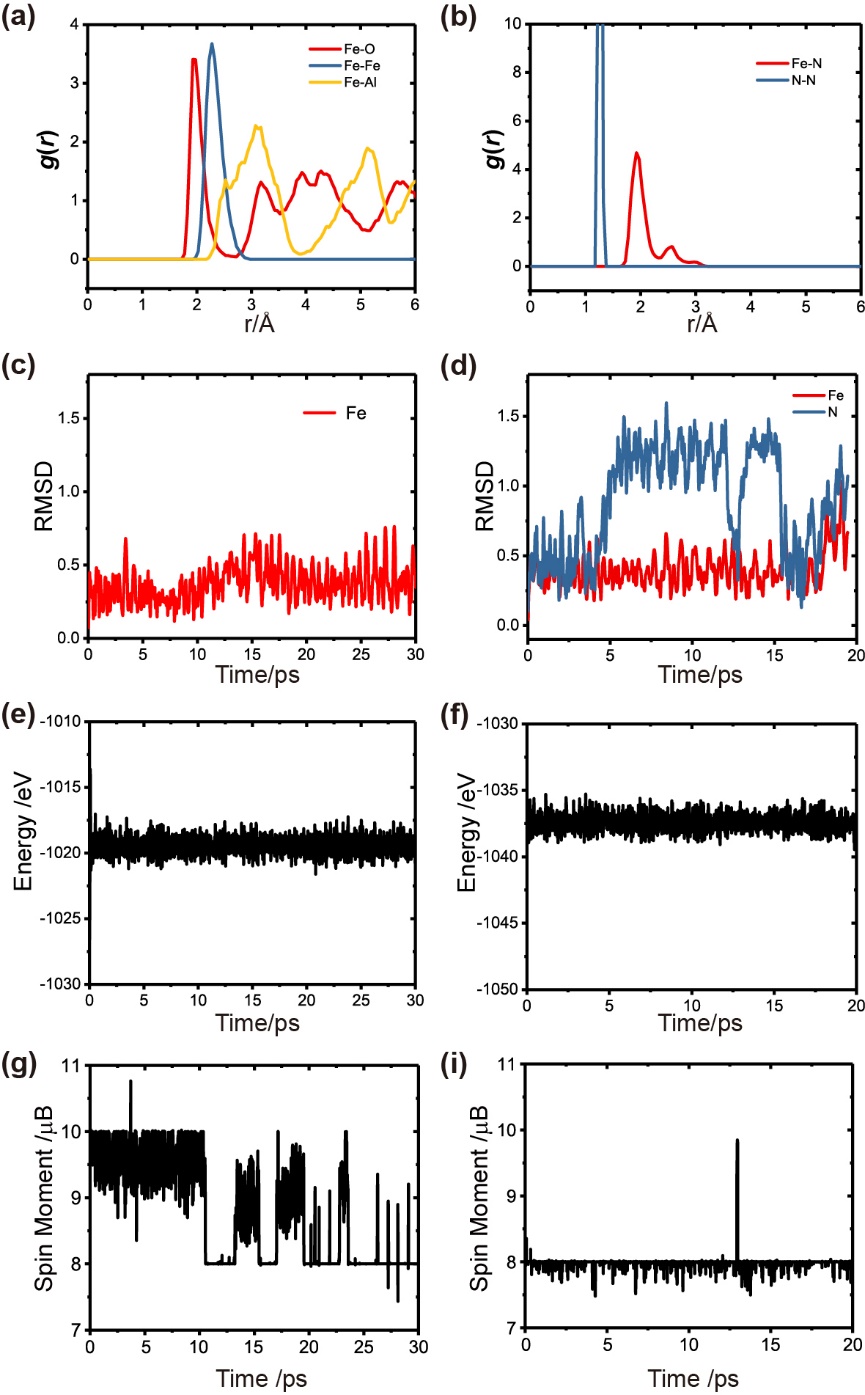


**Supplementary Figure 5.** AIMD simulations. Fe_3_/θ-Al_2_O_3_(010) (left) and Fe_3_N_2_/θ-Al_2_O_3_(010) (right). Radial distribution functions (RDFs) of (a) Fe-O, Fe-Al, Fe-Fe for Fe_3_/θ-Al_2_O_3_(010) and (b) Fe-N, N-N for Fe_3_N_2_/θ-Al_2_O_3_(010); (c, d) root-mean-square deviation (RMSD) of Fe and N; (e, f) fluctuations of potential energy with time; (g, i) spin moment change with time.


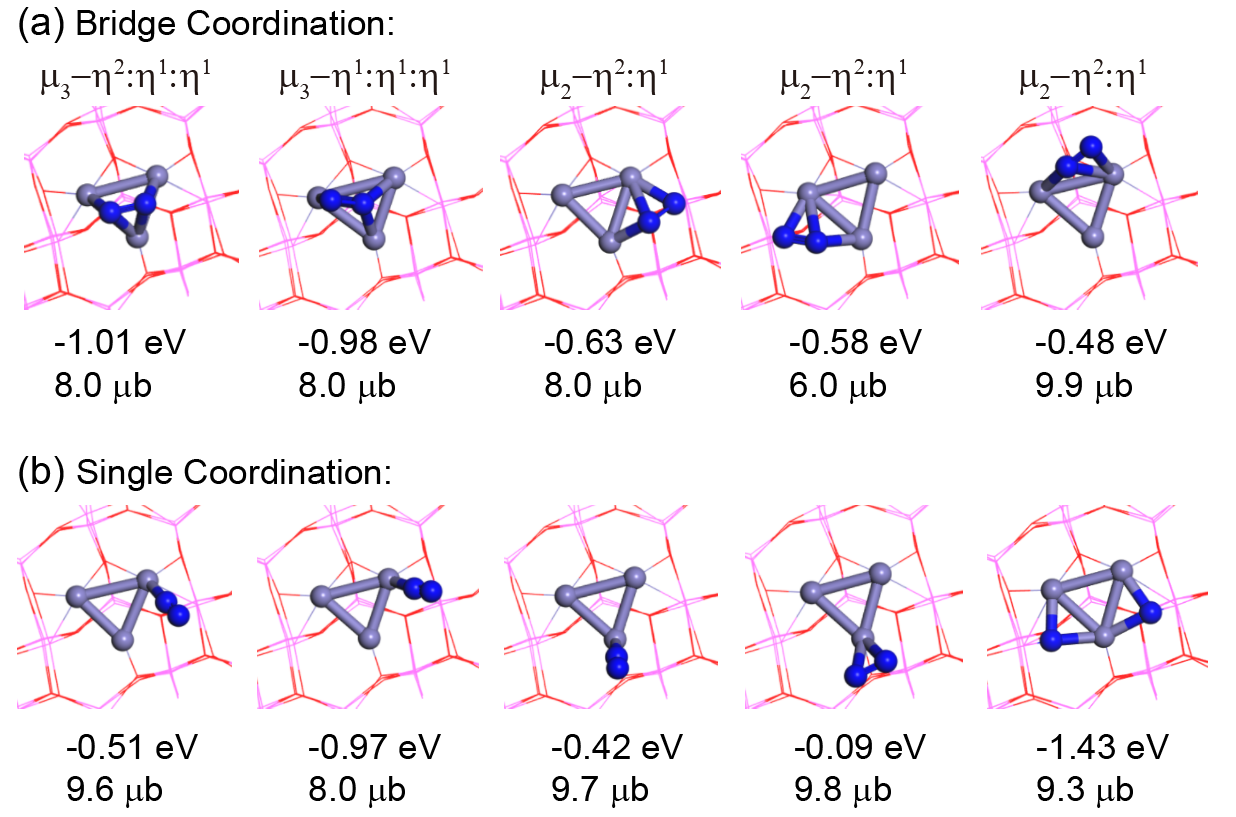


**Supplementary Figure 6.** Optimized configurations, spin and energies. Bridging and single-atom coordination, corresponding spin moments and energies of N_2_ adsorption on Fe_3_/θ-Al_2_O_3_(010).


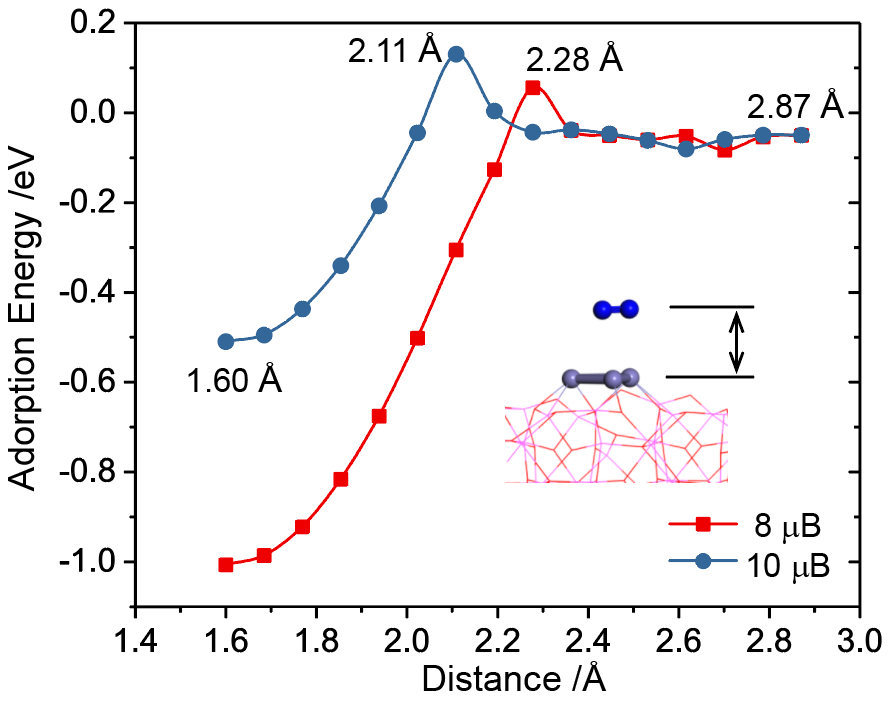


**Supplementary Figure 7.** The binding energy curves for adsorption of N_2_ to Fe_3_/θ-Al_2_O_3_(010) with spin moment of 8 μB and 10 μB. When an N_2_ molecule approaches the supported Fe_3_ center, physisorption occurs first with weak adsorption energy. Although the energies of the two surfaces are nearly the same when N_2_ is far from Fe_3_ center, by overcoming a tiny barrier of around 0.2 eV, the energy decreases to around -1.01 eV on 8 μB surface but only to around -0.51 eV on 10 μB surface.


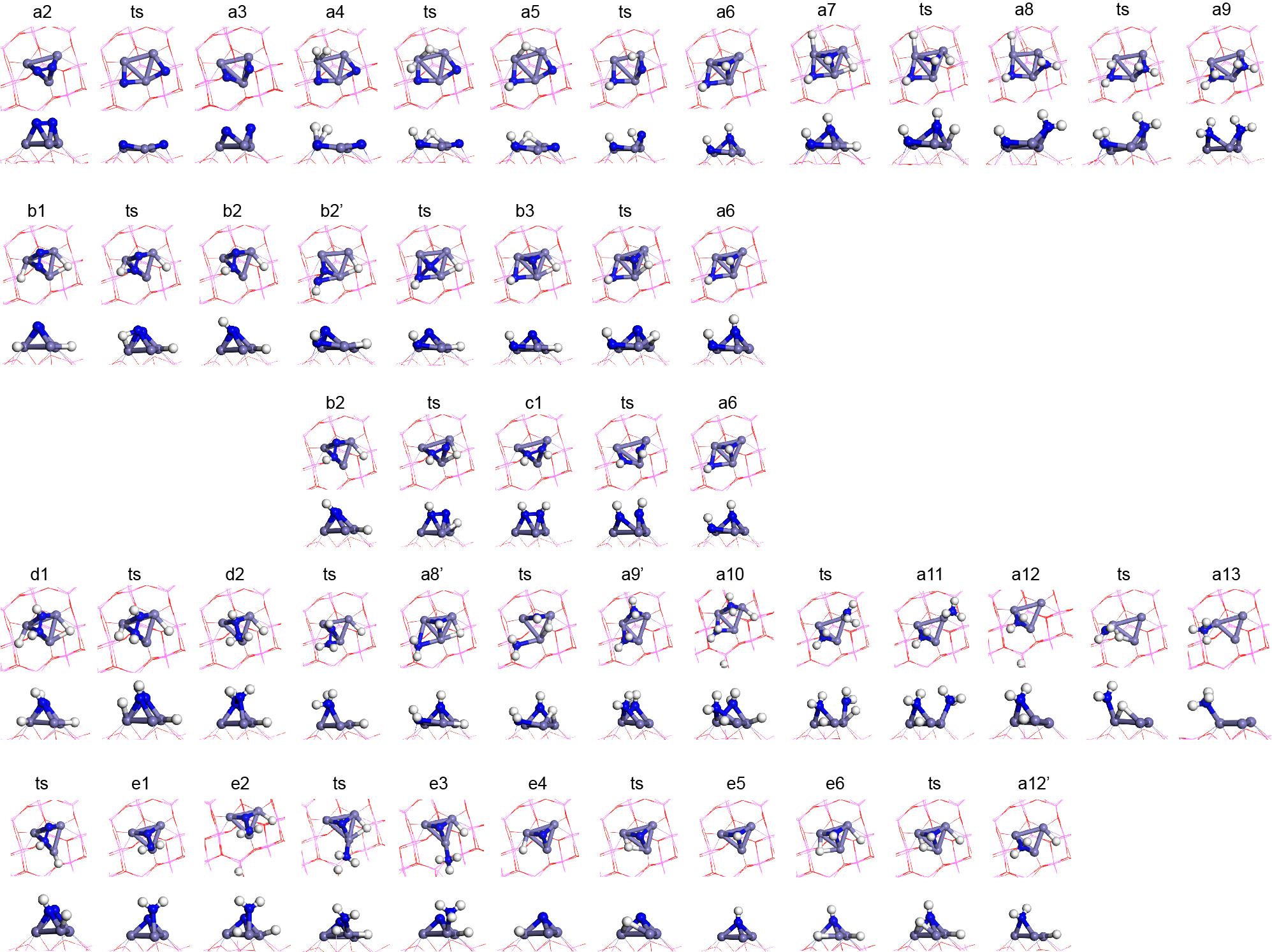


**Supplementary Figure 8**. Optimized atomic configurations for every step in Figure 3d.


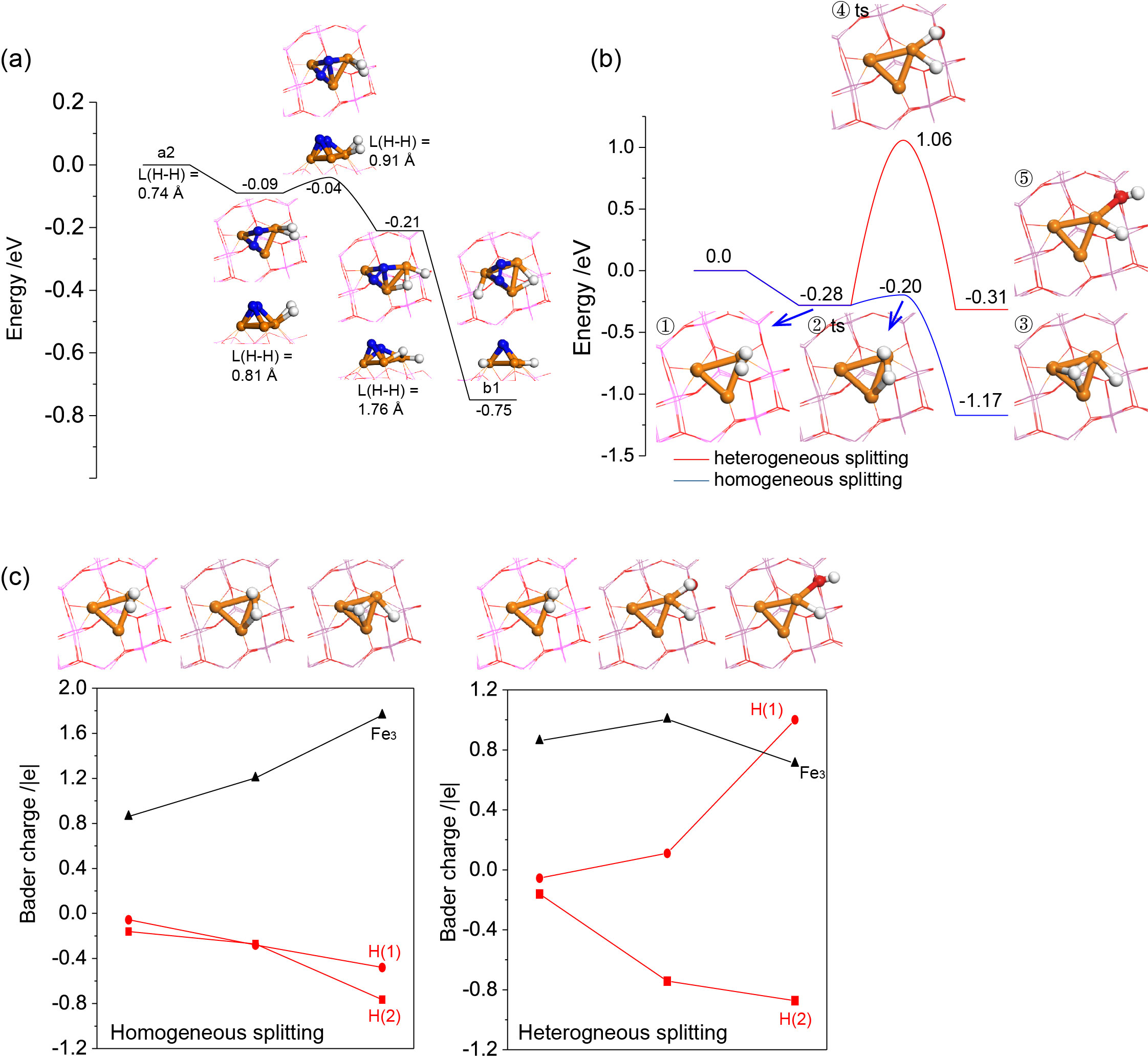


**Supplementary Figure 9**. Energy diagram, charges and barriers. (a) DFT energy diagram for H_2_ dissociative adsorption on the pre-N_2_ occupied Fe_3_ cluster. (b) Homogeneous (blue) and heterogeneous (red) splitting pathways at the Fe_3_ cluster and Fe_3_/Al_2_O_3_ interface. (c) Bader charge changes of the two H and the Fe_3_ cluster during the H_2_ dissociative adsorption step. The barrier for homogeneous splitting is less than 0.1 eV, which is much lower than that for the heterogeneous splitting (1.34 eV), because the interface oxygen is coordinated with two Al and one Fe, and does not favor another proton. The homogeneous splitting of H_2_ leads to oxidation of Fe_3_ cluster and two hydrides that are responsible for the reduction of N_2_.


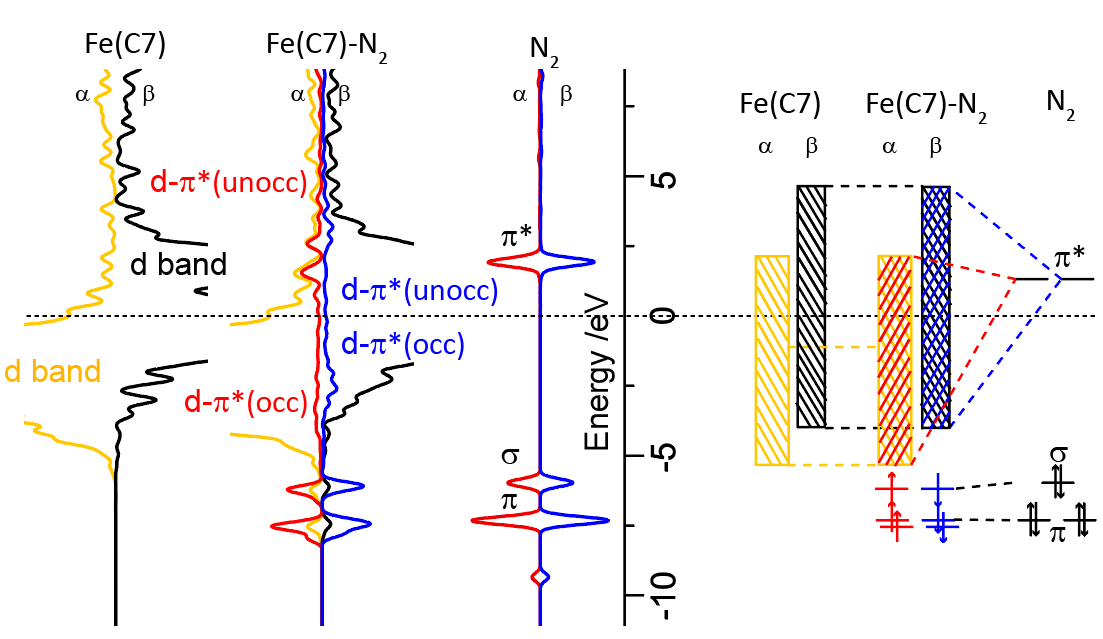


**Supplementary Figure 10**. Projected density-of-states and energy levels. Left: Projected density-of-states (pDOS) of the seven Fe atoms at the C7 site of Fe(221) surface, Fe(211)N_2_, and N_2_ molecules. Right: corresponding schematic illustration of the energy levels from the pDOS graph.

**Supplementary Table 1.** Orbital types, transformation properties, and bonding types of the radial (r), vertical (v), and tangential (t) d-type group orbitals of Fe atoms in Fe_3_ clusters under D_3h_ symmetry

| Orbital type | Irreducible  representations (D_3h_) | Bonding type |
| --- | --- | --- |
| Γd_x_^2^_-y_^2^ | a_1_’  e’ | σ_r_  σ_r_* |
| Γd_z_^2^ | a_1_’  e’ | δ_v_  δ_v_* |
| Γd_xz_ | a_2_’’  e’’ | π_r_  π_r_* |
| Γd_yz_ | a_1_’’  e’’ | π_t_*  π_t_ |
| Γd_xy_ | a_2_’  e’ | σ_t_*  σ_t_ |


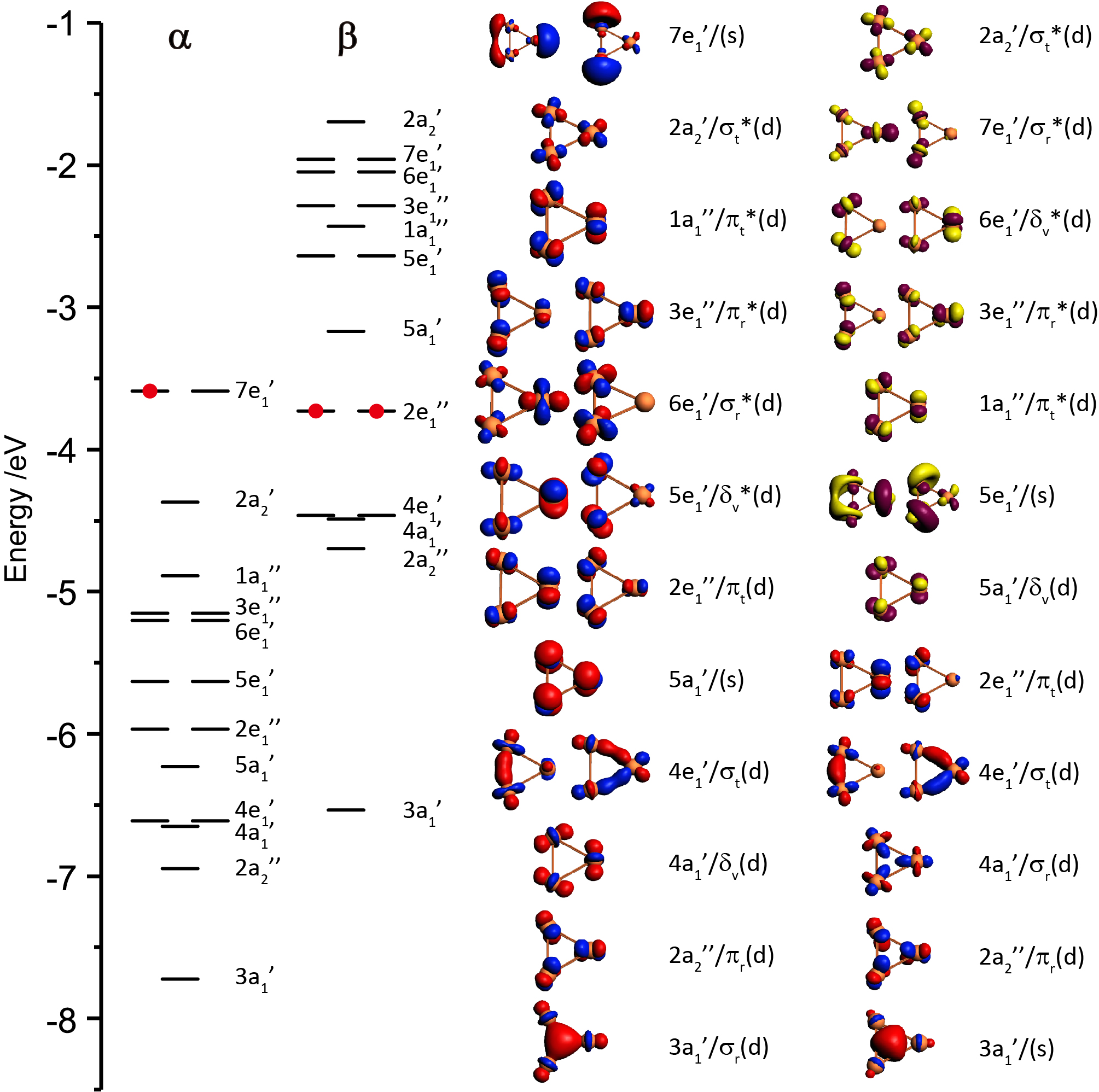


**Supplementary Figure 11.** Kohn–Sham MO analysis of the isolated Fe_3_ fragment with D_3h_ configuration based on SR-ZORA PBE/TZ2P calculations. 10 types of MOs from D_3h_ Fe_3_ molecule are defined: σ_r_, σ_t_, σ_r_*, σ_t_*; π_r_, π_t_, π_r_*, π_t_*; and δ_v_, δ_v_*, with the asterisk indicating the antibonding MOs. Here the cluster-skeletal MOs are classified as radial (r), tangential (t), vertical (v) according to the orientation of the AOs in a local coordinate system (LCS) relative to the Fe_3_ triangle. The electronic configuration is (3a_1_’)^2^(2a_2_’’)^2^(4a_1_’)^2^(4e_1_’)^4^ (2e_1_’’)^4^(5a_1_’)^1^(5e_1_’)^2^ (6e_1_’)^2^(3e_1_’’)^2^(1a_1_’’)^1^(2a_2_’)^1^(7e_1_’)^1^. The energy levels of Fe_3_ α-orbitals are much lower than those of the β−orbitals due to large exchange stabilization energy of the α−electrons. Such supported Fe_3_ is of comparative interest for nitrogen activation, since it has low charge state and centralized spin moment with exposed trimer center to interact with N_2_.


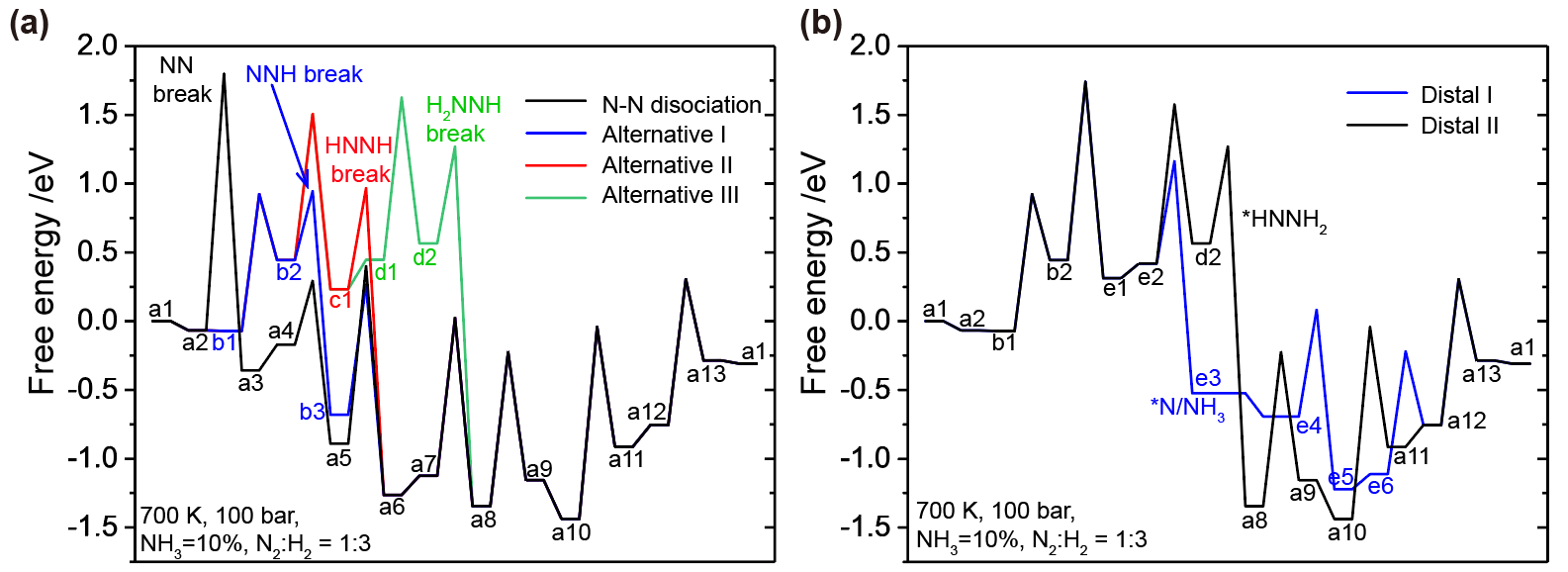


**Supplementary Figure 12**. Gibbs free energy diagram for ammonia synthesis on the Fe_3_/θ-Al_2_O_3_(010). (a) *N_2_ dissociative mechanism, and three pathways of associative mechanism with N-N bond dissociation at *NNH, *HNNH, and *HNNH_2_ intermediates by the alternating hydrogenation route. (b) Two pathways of *N_2_ associative mechanism by distal hydrogenation route. Reaction conditions and parameters are: T = 700 K, P = 100 bar, H_2_:N_2_ ratio = 3:1, and NH_3_ conversion is fixed at 10 %.


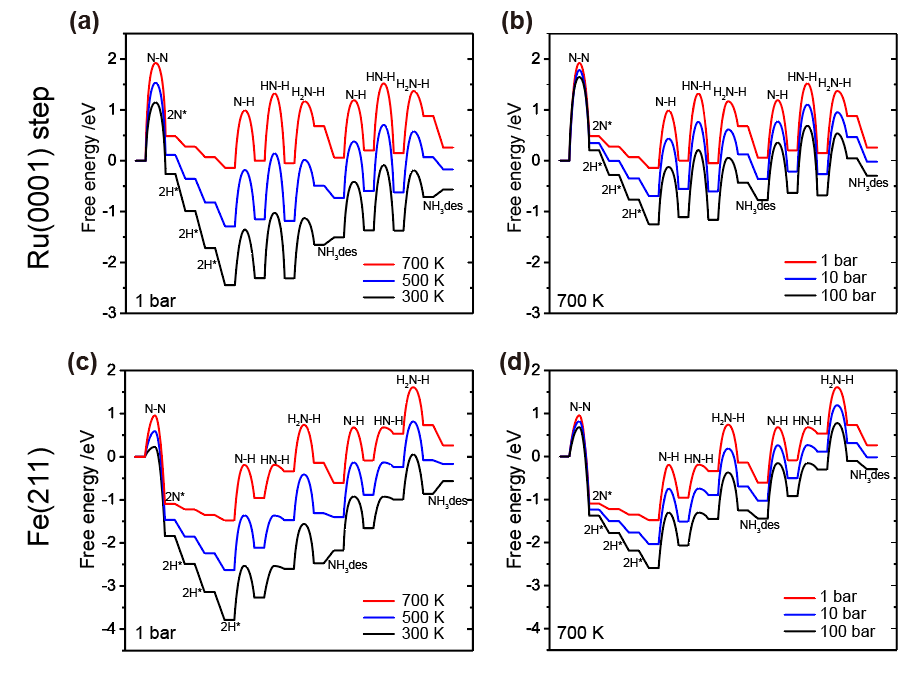


**Supplementary Figure 13**. Gibbs free energy diagram for ammonia synthesis on the (a, b) Ru(0001) step, and (c, d) Fe(211) surface. Reaction conditions and parameters are: H_2_:N_2_ ratio = 3:1 and NH_3_ conversion is fixed at 10 %.


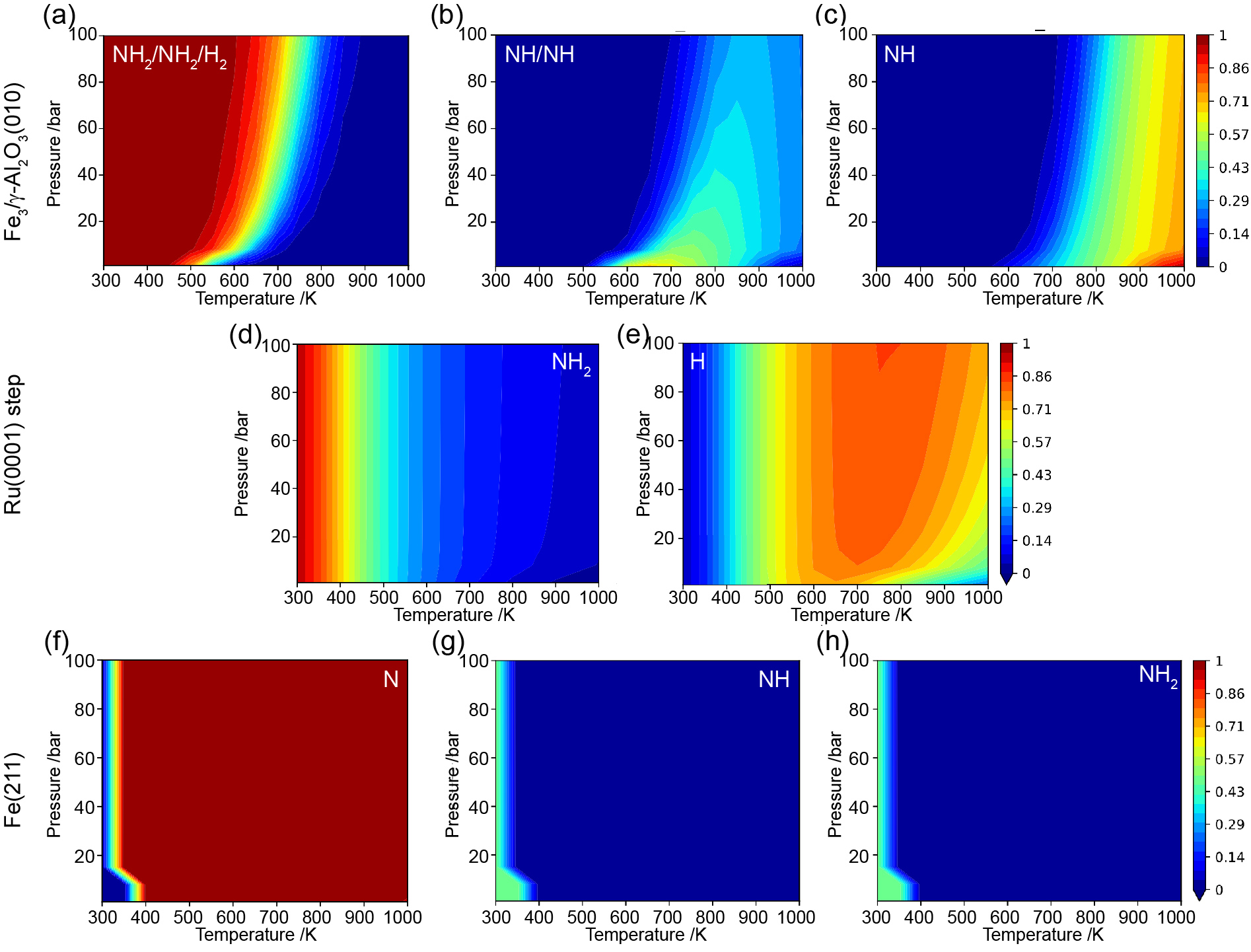


**Supplementary Figure 14**. Coverage for surface species on (a-c) Fe_3_/θ-Al_2_O_3_(010), (d, e) Ru (0001) step, and (f-h) Fe(211) surface mapped with pressure (1 – 100 bar) and temperature (300 – 1000 K). Reaction conditions and parameters are: H_2_:N_2_ ratio = 3:1 and NH_3_ conversion is fixed at 10 %.


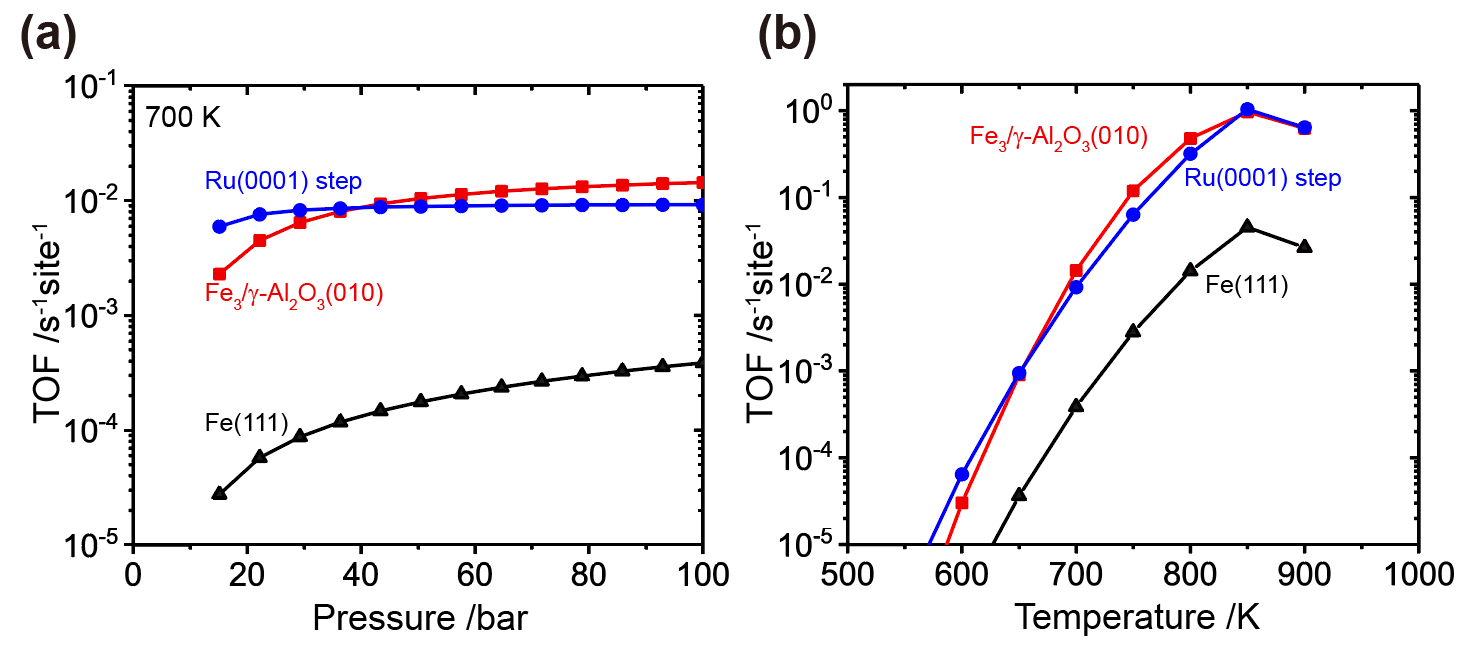


**Supplementary Figure 15**. Calculated turnover frequencies. (a) TOFs per site of ammonia synthesis over the Fe_3_/θ-Al_2_O_3_(010), Ru (0001) step, and Fe(211) surface at different pressure (1 – 100 bar) with a fixed temperature of 700 K. (b) TOFs at different temperature (300 – 1000 K) with a fixed pressure of 100 bar. Reaction conditions and parameters are: H_2_:N_2_ ratio = 3:1 and NH_3_ conversion is fixed at 10 %. TOFs are calculated using ideal gas and harmonic vibrational approximation.
