# Supplementary material for: Heterogeneous Fe3 single-cluster catalyst for ammonia synthesis via an associative mechanism
Source: Nat Commun. 2018 Apr 23;9:1610. doi: 10.1038/s41467-018-03795-8 (PMC5913218; doi:10.1038/s41467-018-03795-8)
Supplement: Supplementary file 2 — Descriptions of Additional Supplementary Files [file 41467_2018_3795_MOESM2_ESM.pdf]

Descriptions of Additional Supplementary Files

File Name: Supplementary Movie 1
Description: AIMD trajectory showing H_2 dissociation on supported Fe_3 cluster
